# Anxiety and depression among Greek men with primary erectile dysfunction and premature ejaculation

**DOI:** 10.1186/s12991-015-0074-y

**Published:** 2015-10-29

**Authors:** Iraklis Mourikis, Marianthi Antoniou, Efi Matsouka, Eleni Vousoura, Chara Tzavara, Chrysa Ekizoglou, George N. Papadimitriou, Nikos Vaidakis, Iannis M. Zervas

**Affiliations:** Department of Psychiatry, Eginition Hospital, Athens University Medical School, 72-74 Vas. Sophias Ave., 11528 Athens, Greece; Sexual Disorders Clinic, First Department of Psychiatry, Eginition Hospital, 74 Vas. Sofias Ave, 11528 Athens, Greece

**Keywords:** Sexual dysfunction, Erectile dysfunction, Premature ejaculation, STAI, Anxiety, Depression

## Abstract

**Background:**

Erectile dysfunction (ED) and premature ejaculation (PE) are the two most prevalent sexual disorders among males associated with significant distress and impairment in quality of life. The aim of this study was to investigate the prevalence of anxiety and depression symptoms among patients with primary ED and PE.

**Methods:**

A sample of 57 men (ED = 31; PE = 26) were compared to 25 male outpatients with anxiety disorder (AD) and 25 healthy controls. Principal assessment measures included the State-Trait Anxiety Inventory (STAI) and the Beck Depression Inventory (BDI).

**Results:**

Greater levels of STAI state anxiety were reported among the ED, PE, and AD groups as compared to healthy controls. In contrast ED and AD groups scored higher than controls on the STAI trait anxiety and BDI, but PE scores were not different from healthy controls in both measures.

**Conclusions:**

The study findings suggest that both primary ED and PE are conditions associated with significant state anxiety; however, PE appears to be less associated with trait anxiety and depression compared to ED, a finding that corroborates the recent acknowledgement of PE as a more biologically based condition. Limitations and potential clinical implications are also discussed.

## Background

Sexual dysfunction is characterized by disturbances during any stage of the sexual response cycle. It is a common, yet debilitating, condition affecting both men and women at some point in their lives and preventing them from experiencing satisfaction from the sexual activity [1]. Among men, the two most prevalent sexual complaints are erectile dysfunction (ED) and premature ejaculation (PE) [2].

ED is defined as persistent inability to achieve and maintain an erection sufficient for satisfactory sexual performance [3]. It is estimated to affect up to 52 % of men globally, of whom 5–20 % experience moderate to severe ED symptoms [4–6]. PE is defined as persistent ejaculation with minimal sexual stimulation before or soon after penetration, in which the individual has minimal voluntary control over [7]. It is a highly common condition, with prevalence rates reported in epidemiological studies as high as of 30 % [6, 8–12]. Both disorders can be profoundly disabling, causing long-lasting adverse effects on self-image, significant interpersonal and intimacy difficulties [3, 13–15], and mental health problems [13–15].

It has long been thought that sexual disorders, including ED and PE, are primarily due to psychological factors. Anxiety and depression in specific are considered to play a major role in the development and maintenance of problems related to sexual functioning [16, 17]. Several studies have identified an association between anxiety and ED [18–21], as well as anxiety and PE [22, 23], although some studies have found that anxiety may in fact facilitate sexual arousal [17, 24]. A strong association exists between depression and ED and the strength of that relationship increases with the severity of depression [25, 26]. Depression has also been linked to PE [9, 27], with recent findings showing that this relationship increases with the duration of PE [28].

Recent studies have pointed out that ED and PE may be differentially affected by anxiety and depression symptoms. For instance, genetic rather than psychological factors are increasingly recognized as important contributors to the development of PE [29], while performance and free-floating anxiety appear to play a crucial role on the development and sustenance of ED [18]. To our knowledge, there are no studies that directly compare patients with ED and PE on anxiety and depression symptoms. In addition, very few studies have investigated sexual disorders among Greek males [30].

The study attempts to fill this important gap in the literature by investigating whether ED and PE patients differ in terms of state and trait anxiety and depression symptoms. To make meaningful comparisons between these two groups, two additional groups were included; one of healthy controls and one group of outpatients with anxiety disorder. Based on prior research evidence, we hypothesized that patients with ED and PE will report STAI and BDI scores higher than healthy controls, but comparable to AD patients; we also hypothesized that there will be differences in trait (but not state) STAI and BDI scores between ED and PE patients.

The clarification of the relationship between male sexual disorders and anxiety and depression is essential for proper assessment and treatment of ED and PE; by understanding better the role of anxiety and depression in the development and maintenance of male sexual disorders, clinicians will be able to design and implement interventions tailored to their patients’ specific needs.

## Methods

### Sample

The study was approved by the Ethics Committee of Eginition Hospital in Athens, Greece. The total sample consisted of 107 male participants: 57 outpatients diagnosed with sexual dysfunction, 25 outpatients diagnosed with anxiety disorders, and 25 healthy controls, described in detail below.

*Sexual dysfunction group* Participants in the sexual dysfunction group were recruited from the Outpatient Specialty Clinic for Sexual Disorders of Eginition Hospital in the Department of Psychiatry of the Medical School of Athens between September 2010 and July 2014. The clinic is part of a large, inner city psychiatric hospital and treats a diverse patient population seeking help for a wide range of psychosexual problems, commonly referred to the clinic by either a medical professional (e.g., urologist, general practitioner) or telephone helplines offering support and counseling for sexual problems. Eligible participants in this study were adult, heterosexual men with an exclusive diagnosis of primary (lifelong) ED or PE according to the Diagnostic and Statistical Manual of Mental Disorders—4th Edition (DSM-IV-TR) criteria [7], confirmed by the Mini International Neuropsychiatric Interview (MINI) [31].

Prior to participation in the study, all eligible participants had undergone a comprehensive andrological diagnostic workup, which included obtaining detailed medical history, physical examination, and hormonal evaluation to rule out organicity of reported sexual problems. Organic ED was ruled out by confirming the presence of morning or situational (e.g., masturbatory) erections. Other exclusion criteria were (a) medical conditions interfering with sexual function; (b) medications affecting sexual function administered within 30 days before screening; (c) alcohol or substance abuse; (d) presence of major psychiatric disorder and/or suicide risk. Fifty-seven patients meeting the above criteria agreed to enroll in the study by signing an informed consent; 31 (54 %) were diagnosed with primary ED and 26 (46 %) with primary PE.

*Anxiety disorder group* The anxiety group included 25 adult male outpatients who were evaluated in the Behavioral Treatment Unit at the Department of Psychiatry of the University of Athens Medical School and met criteria for an anxiety disorder according to DSM-IV-TR [7]. Diagnoses included obsessive compulsive disorder (*N* = 8), panic disorder (*N* = 14), and generalized anxiety disorder (*N* = 3). Participants had to be between ages 18 and 65 with no diagnosis of sexual disorder as assessed by the initial psychiatric assessment conducted at the clinic and further confirmed by the International Index of Erectile Function Questionnaire (IIEF) [32]. None of the participants received pharmacological or psychosocial treatment at the time of data collection, as they had just completed evaluation and were awaiting treatment placement.

*Control group* The control group included 25 healthy men who were opportunistically recruited from friends or relatives of the hospital personnel (75 % participation rate). Exclusion criteria were diagnosis of sexual dysfunction or any psychiatric disorder as assessed by the IIEF [32] which is further described below.

### Procedure and measures

The study was conducted at Eginition Hospital between 2010 and 2014. The study was in accordance with the protocol and ethical principles stated in the Declaration of Helsinki and approved by the Ethics Committee of Eginition Hospital. Following a complete description of the study, written informed consent was obtained from all participants. Information about age, marital status and educational level of the participants was obtained through a self-report demographic questionnaire. The following standardized scales were administered to measure key concepts for the study.

*State*-*trait anxiety inventory* (STAI) [33]. Anxiety symptoms were measured with STAI, a self-administered scale consisting of 40 questions rated on a four-point Likert-type scale from “Almost Never” to “Almost Always” with higher scores indicating greater anxiety. STAI provides scores for two 20-item subscales, one for state anxiety, i.e., the level of anxiety experienced at the time of assessment, and one for trait anxiety, i.e., the more general, pervasive and longstanding tendency for experiencing anxiety. The STAI (Form Y) has been translated in Greek and validated in the Greek population with adequate psychometric properties [34].

*Beck depression inventory* (BDI) [35]. Depression symptoms were measured with the BDI, which is a self-administered 21-item questionnaire of neurovegetative and cognitive–emotional symptoms of depression. BDI is used both for the screening of depression and assessment of severity of depressive symptomatology. BDI has been translated and validated for use in the Greek population [36].

*International Index of Erectile Function Questionnaire* (IIEF) [32]. Sexual function was measured with IIEF, a brief, self-administered scale that consists of 15 questions assessing five domains of male sexual function: erectile function (six items), orgasmic function (two items), sexual desire (two items), intercourse satisfaction (three items), and overall satisfaction (two items). Scores are calculated for the 5 domains as well as for the Total Index score, with higher scores indicating better sexual functioning.

*Life events* Life events were assessed by a short, self-administered questionnaire especially devised for the present study. The questionnaire contained a list of 18 life events commonly reported in this population, covering the areas of personal, family, work, and social life, which have occurred over the last year.

### Statistical analysis

Continuous variables are presented with mean and standard deviation (SD) or with median and interquartile range (IQR). Qualitative variables are presented with absolute and relative frequencies. Fisher’s exact test was used for the comparison of proportion. Analysis of variance (ANOVA) was performed to compare age between the four study groups. For the comparison of life events between the four groups the Kruskal–Wallis test was used. Univariate linear regression analyses were conducted to explore differences in state, trait, and depression between the four study groups. Afterwards, multiple linear regression analyses were performed to explore the differences found in univariate analysis after adjusting for age, family status and number of life events that the subjects had experienced. Regression coefficients and standard errors were computed from the results of the linear regression analyses. Diagnostics for regression models were performed to check if the conditions for regression had been met with the residuals of each model being normally distributed and their variance being constant. Interactions of variables in the models were not significant. All reported *p* values are two tailed. Statistical significance was set at *p* <0.05 and analyses were conducted using SPSS statistical software (version 19.0).

## Results

The sample consisted of 107 participants (31 in the ED group, 26 in the PE group, 25 in the AD group, and 25 healthy controls) with mean age 36.3 years (SD = 10.6 years). The four groups were comparable in terms of age and family status. Sample demographic characteristics and number of life events are presented in Table 1. The median number of life events overall, as well as those related to economic issues, was similar between the four groups (*p* > 0.05). Table 2 illustrates the mean scores on STAI and BDI scores for the four study groups.

**Table 1:** Sample characteristics

	Group				*p*
	ED *N* = 31	PE *N* = 26	Controls *N* = 25	AD *N* = 25	
Age, mean (SD)	38.8 (12.9)	37.9 (10.1)	34.0 (6.9)	34.4 (6.2)	0.17*
Family status, *N* (%)					
Married	13 (41.9)	14 (53.8)	6 (24.0)	6 (24.0)	0.13^a^
Unmarried	13 (41.9)	8 (30.8)	10 (40.0)	11 (44.0)	
In relationship	4 (12.9)	3 (11.5)	9 (36.0)	8 (32.0)	
Divorced	1 (3.2)	1 (3.8)	0 (0.0)	0 (0.0)	
Number of life events, median (IQR)	2 (1–4)	2 (1–3)	15 (60.0)	3 (2–3)	0.62**
Number of life events concerning economic issues, median (IQR)	0 (0–2)	0 (0–1)	1 (1–1)	1 (0–1)	0.10**
Number of other than economical life events, median (IQR)	1 (1–2)	1 (1–2)	1 (0–2)	1 (1–2)	0.98**

*ED* erectile dysfunction, *PE* premature ejaculation, *AD* anxiety disorder

* ANOVA

** Kruskal–Wallis test

^a^Fisher’s exact test

**Table 2:** Mean values on STAI, BDI and IIEF dimensions for the four study groups

	ED	PE	Controls	AD
	Mean (SD)	Mean (SD)	Mean (SD)	Mean (SD)
STAI				
State	48.1 (3.9)	47.8 (5.3)	43.8 (6.4)	48.0 (3.7)
Trait	47.8 (5.7)	45.7 (6.7)	44.1 (5.6)	53.5 (6.0)
Depression (BDI)	12.0 (8.5)	10.2 (7.6)	7.5 (6.2)	14.0 (8.7)
Minimal	20 (64.5)	18 (69.2)	21 (84.0)	14 (56.0)
Mild	4 (12.9)	6 (23.1)	3 (12.0)	6 (24.0)
Moderate	6 (19.4)	1 (3.8)	1 (4.0)	4 (16.0)
Severe	1 (3.2)	1 (3.8)	0 (0.0)	1 (4.0)
IIEF				
Erectile function	12.2 (8.2)	21.9 (8.1)	27.6 (2.7)	24.5 (5.3)
Orgasmic function	5 (3.9)	7.4 (3.4)	9 (1.8)	8.2 (2.3)
Sexual desire	6.3 (2.4)	8 (2.2)	8.4 (1.5)	7.2 (1.7)
Intercourse satisfaction	4.5 (4.2)	7.3 (4.3)	13.1 (2.0)	9.8 (3.6)
Overall satisfaction	4.1 (2.2)	5.8 (2.1)	8.5 (1.6)	5.6 (2.5)
Total index	32.1 (18.0)	49.3 (16.5)	66.6 (7.0)	55.3 (12.6)

*ED* erectile dysfunction, *PE* premature ejaculation, *AD* anxiety disorder

Results from univariate and multiple regression models investigating group differences in state/trait and BDI scores are presented in Table 3. Both univariate and multiple analyses showed significantly greater levels of state anxiety in the ED, PE, and AD groups as compared to healthy controls. No significant differences in state anxiety scores were found between the ED and PE groups, or between the AD group versus ED and PE groups, suggesting that all three clinical groups endorsed equivalent levels of state anxiety.

**Table 3:** Results from univariate and multiple regression analysis for differences in STAI and BDI between the study groups

	State Anxiety		Trait Anxiety		Depression (BDI)	
	Unadjusted	Adjusted^b^	Unadjusted	Adjusted^b^	Unadjusted	Adjusted^b^
	*β* (SE)^a^	*β* (SE)^a^	*β* (SE)^a^	*β* (SE)^a^	*β* (SE)^a^	*β* (SE)^a^
Controls. *ref.*						
ED	4.31 (1.19)**	3.58 (1.23)**	3.76 (1.61)*	4.00 (1.76)*	4.55 (2.13)*	4.74 (2.13)*
PE	4.01 (0.72)**	3.75 (0.72)**	1.60 (1.70)	2.06 (1.76)	2.76 (2.22)	2.36 (2.27)
AD	4.20 (1.41)**	4.30 (1.51)**	9.44 (1.70)***	8.94 (1.72)***	6.56 (2.22)**	5.08 (2.22)*
PE. *ref.*						
ED	1.03 (1.34)	1.05 (1.37)	2.16 (1.61)	1.94 (1.57)	1.79 (2.13)	0.90 (2.04)
AD. *ref.*						
ED	0.10 (1.35)	0.25 (1.43)	−5.68 (1.61)**	−4.94 (1.61)**	−2.01 (2.13)	−1.82 (2.08)
PE	−0.20 (1.40)	−0.18 (1.45)	−7.84 (1.70)***	−6.88 (1.66)***	−3.80 (2.22)	−2.72 (2.15)

*ED* erectile dysfunction, *PE* premature ejaculation, *AD* anxiety disorder, *ref* reference category

^a^Regression coefficient (standard error)

^b^Adjusted for age, family status and number of life events

* *p* < 0.050; ** *p* < 0.010; *** *p* < 0.00

Regarding the trait anxiety scores, while significant differences were observed between healthy controls and the ED group (*p* < 0.05) and the AD group (*p* < 0.001), there was no difference between controls and the PE group (*p* > 0.05). Both ED and PE groups reported significantly lower trait anxiety scores than the AD group. Participants in the PE group scored lower on the trait anxiety scale than their ED counterparts, but the difference did not reach statistical significance. With regard to depression symptoms, both AD and ED groups scored significantly higher on the BDI compared to healthy controls. A trend towards higher BDI scores than controls was noticed for the PE group. No significant BDI score differences were found between AD group and ED or PE groups.

## Discussion

The aim of this study was to investigate the prevalence of state and trait anxiety and depression in primary ED and PE. To draw meaningful conclusions, we included a group of anxious patients and a group of healthy controls.

Our results provide evidence for the differential role of anxiety and depression in primary ED and PE. Both sexual dysfunction groups endorsed higher state anxiety scores compared to healthy controls, but lower than patients with ADs. Interestingly, the results for the trait anxiety were different. As expected, both sexual dysfunction groups reported lower trait anxiety. However, ED patients had significantly higher scores than healthy controls, unlike the PE group which scored comparably to the control group. Similarly, our results showed that ED patients scored higher than controls in depression as measured by the BDI, while scores among the PE group were not different from controls.

Our results on state anxiety, indicating that all clinical groups (ED, PE, and AD) had significantly higher state anxiety compared to healthy controls, are in agreement with previous research findings. For instance, a recent study with 882 ED and PE patients found that both PE and ED groups were associated with excess of free-floating anxiety [18]. While the levels of state anxiety are equivalent among the three groups, the type of situational anxiety triggered in each clinical group is hypothesized to be different. In ADs, situational anxiety arises as a response to selective attention to threat information coupled with a distorted cognitive processing of the situation (i.e., catastrophic misappraisal). For example, in panic disorder, attention is selectively directed toward somatic symptoms which are assessed as dangerous, while in generalized anxiety disorder, attention is focused on external danger triggering ruminative thoughts regarding the impending disaster and its potential prevention [37].

In sexual dysfunctions, anxiety, and stress reactions in general are viewed as cognitive interferences that distract attention from sexual stimuli thus inhibiting sexual arousal. The situational stress in sexual dysfunctions may take the form of “performance anxiety” [38, 39]. This anxiety appears to interfere with sexual functioning through altering the neural substrate of sexual behavior, further contributing to the maintenance of sexual dysfunctions. The performance anxiety affects the normal sexual functioning through selective attention [40–42].

Erection concern thoughts reduce sexual arousal and intervene with normal sexual functioning [43]. These thoughts are automatic in nature and focus on sexual performance (e.g., “my penis is not responding” “why can’t it work”?). These cognitions are accompanied by selective focus on erectile response and the negative consequences of a possible sexual “failure”, thus impairing the processing of sexual stimuli, reducing the frequency of sexual fantasies and increasing negative emotions (e.g., sadness, shame) [44]. The selective focus on sexual performance and the possible negative consequences of failure, combined with the absence of sexual thoughts and depressed mood further reinforce the cycle of sexual difficulties and mood problems [38, 43, 44].

Moreover, Nobre [44] focuses on a particular belief that seems to play a dominant role in erectile dysfunction, namely the “macho myth” (i.e., myth of the male role), which seems to result in rigid beliefs and expectations about sexual performance. Examples of such rigid beliefs include “I have to have frequent sexual activities”, “I need to be able to satisfy any partner”, or “it is necessary to maintain my erection under any circumstance”. Such rigid attitudes and expectations regarding sexual performance render men more susceptible to have a negative schema of inadequacy triggered when exposed to an “unsuccessful” sexual experience [44].

Clinical practice reveals a plethora of negative thoughts that could lead to state anxiety and subsequent sexual dysfunction, such as feeling “trapped”, or fearing that the partner “will break up” with or “cheat on” the individual. In addition, several other factors play an important role in sexual dysfunction, including emotional expression and proximity in the paternal family, presence of potentially traumatic sexual experiences, and religious beliefs [44]. Also, social messages through media can influence and shape the modern perception of sexuality and prescribed male and female sexual roles, often promoting a “vulnerable” sexual culture [45].

Our study findings regarding trait anxiety revealed a different pattern. Not surprisingly, trait anxiety was found to be significantly higher among individuals with anxiety disorders compared to all three other groups. Indeed, the strong association between anxiety disorders and trait anxiety is well supported in the literature [46]. However, the finding that ED patients—but not PE—scored higher in trait anxiety compared to healthy controls has interesting implications. Stated differently, patients with PE had comparable trait anxiety scores to healthy controls. Taken together, these findings are in agreement with contemporary research evidence suggesting that organic, neurobiological rather than psychological factors are implicated in the development of primary PE [47, 48].

A similar pattern was observed for depression symptoms. Again, AD patients scored the highest among the three clinical groups, whereas ED but not PE patients scored significantly higher on the BDI than controls. This result echoes previous research findings showing that depression is a risk factor for ED but not for PE [49, 50]. Several studies have identified a high comorbidity between depression and ED [51–56] but the directionality of this relationship has yet to be determined [57, 58]. It may be that depression symptoms hinder sexual arousal and impair erectile function, or vice versa; the presence of erectile difficulties may trigger low mood building up to the development of depressive syndrome [51, 52]. On the contrary, patients with PE are less likely to experience depression symptoms, a finding that further supports the neurobiological nature of the disorder possibly influenced by hereditary factors [44, 59].

Of note, the average number of life events involving economic problems and the average number of all other life events did not differ significantly among the four groups. This finding suggests that, in the present study, economic problems were not a risk factor for sexual dysfunction. Absence of relationship between life events (financial) and PE corroborates previous findings [6]. However, this finding must be interpreted with caution, as data collection of this study took place amidst a period of significant economic hardship due to the Greek financial crisis that affected the majority of the Greek population. Thus, the lack of significant relationship between financial stressors and sexual dysfunction may be attributable to the broader economic context in Greece.

### Limitations

The study has several limitations that merit acknowledgment. The most obvious limitation is the cross-sectional design of the study that does not allow an investigation of causality. Any causal relationships between anxiety and sexual dysfunctions must be explored in prospective studies. Moreover, all measurement tools employed in this study were self-report in nature. Another limitation pertains to the clinical sample of the study that may limit the study’s generalizability to the general population. Finally, our study focused on primary ED and PE patients and thus our results may not be generalizable to patients with sexual dysfunctions acquired later on in their lives. Among the strengths of this study is that it investigates an important subject largely ignored in the literature and it is conducted in the largely understudied population of White Greek patients.

### Implications

The significant clinical differences between ED and PE in terms of anxiety and depression suggest that these two sexual disorder groups may warrant different types of treatment. Compared to ED, PE appears to be less vulnerable to mood and anxiety symptoms and possibly more biologically determined. These findings, which nevertheless await to be confirmed in larger and more robust future studies, may have considerable implications for clinical practice. Mental health practitioners working with male sexual disorders may need to conduct thorough assessment and tailor their treatment plan accordingly to match the different needs of these two groups. Patients with ED may benefit from interventions targeted on modifying core schemas (i.e., “failure”) and beliefs (i.e., “macho myth”) accompanying the disorder. Another effective psychosocial intervention is stress management; a recent study with 31 Greek men with a recent diagnosis of ED found that an 8-week stress management program was effective as an adjunct to tadalafil for the treatment of ED [30]. In contrast, in the case of PE, practitioners should place greater emphasis on neurophysiological contributors of the disorder and determine when it is necessary to refer patients to specialized care.

## Conclusion

To conclude, individuals with primary ED and PE showed comparable levels of state but not trait anxiety or depression symptoms. Results provide support for the central role of neurobiological and genetic factors over psychological factors as the underlying causal mechanism of PE and point towards a differential treatment of the disorders.

## Abbreviations

ED: erectile dysfunction
PE: premature ejaculation
AD: anxiety disorders

## References

[1] Rosen RC (2000). Prevalence and risk factors of sexual dysfunction in men and women. Curr Psychiatry Rep.

[2] Hatzimouratidis K, Amar E, Eardley I, Giuliano F, Hatzichristou D, Montorsi F, Vardi Y, Wespes E (2010). Guidelines on male sexual dysfunction: erectile dysfunction and premature ejaculation. Eur Urol.

[3] Wespes E, Hatzimouratidis K, Eardley I, Al E. Guidelines on male sexual dysfunction. 2013. 10.1016/j.eururo.2010.02.020 20189712

[4] Feldman HA, Goldstein I, Hatzichristou DG, Krane RJ, McKinlay JB (1994). Impotence and its medical and psychosocial correlates: results of the Massachusetts male aging study. J Urol..

[5] Braun M, Wassmer G, Klotz T, Reifenrath B, Mathers M, Engelmann U (2000). Epidemiology of erectile dysfunction: results of the “Cologne Male Survey”. Int J Impot Res.

[6] Laumann EO (1999). Sexual dysfunction in the United States: prevalence and predictors. JAMA J Am Med Assoc.

[7] American Psychiatric Association. Diagnostic and statistical manual of mental disorders, 4th edn., Text Rev. Washington, DC. 2000.

[8] Laumann EO, Nicolosi A, Glasser DB, Paik A, Gingell C, Moreira E, Wang T (2005). Sexual problems among women and men aged 40–80 years: prevalence and correlates identified in the global study of sexual attitudes and behaviors. Int J Impot Res.

[9] Porst H, Montorsi F, Rosen RC, Gaynor L, Grupe S, Alexander J (2007). The premature ejaculation prevalence and attitudes (PEPA) survey: prevalence, Comorbidities, And Professional Help-Seeking. Eur Urol.

[10] Basile Fasolo C, Mirone V, Gentile V (2001). Premature ejaculation: prevalence and associated conditions in a sample of 12,558 men attending the andrology prevention week 2001—a study of the Italian society of andrology (SIA). J Sex Med.

[11] Giuliano F, Patrick DL, Porst H, La Pera G, Kokoszka A, Merchant S, Rothman M, Gagnon DD, Polverejan E (2008). Premature ejaculation: results from a five-country European observational study. Eur Urol.

[12] Patrick DL, Althof SE, Pryor JL, Rosen R, Rowland DL, Ho KF, McNulty P, Rothman M, Jamieson C (2005). Premature ejaculation: an observational study of men and their partners. J Sex Med.

[13] Rowland D, Perelman M, Althof S, Barada J, McCullough A, Bull S, Jamieson C, Ho KF (2004). Self-reported premature ejaculation and aspects of sexual functioning and satisfaction. J Sex Med.

[14] Rowland DL, Patrick DL, Rothman M, Gagnon DD (2007). The psychological burden of premature ejaculation. J Urol.

[15] Symonds T, Roblin D, Hart K, Althof S (2003). How does premature ejaculation impact a man’s life?. J Sex Marital Ther.

[16] Hedon F (2003). Anxiety and erectile dysfunction: a global approach to ED enhances results and quality of life. Int J Impot Res.

[17] Hale VE, Strassberg DS (1990). The role of anxiety on sexual arousal. Arch Sex Behav.

[18] Corona G, Mannucci E, Petrone L, Ricca V, Balercia G, Giommi R, Forti G, Maggi M (2006). Psycho-biological correlates of free-floating anxiety symptoms in male patients with sexual dysfunctions. J Androl.

[19] Petrone L, Mannucci E, Corona G, Bartolini M, Forti G, Giommi R, Maggi M (2003). Structured interview on erectile dysfunction (SIEDY): a new, multidimensional instrument for quantification of pathogenetic issues on erectile dysfunction. Int J Impot Res.

[20] Corona G, Mannucci E, Mansani R, Petrone L, Bartolini M, Giommi R, Mancini M, Forti G, Maggi M (2004). Aging and pathogenesis of erectile dysfunction. Int J Impot Res.

[21] Sugimori H, Yoshida K, Tanaka T, Baba K, Nishida T, Nakazawa R, Iwamoto T (2005). Relationships between erectile dysfunction, depression, and anxiety in Japanese subjects. J Sex Med.

[22] Corona G, Petrone L, Mannucci E, Jannini EA, Mansani R, Magini A, Giommi R, Forti G, Maggi M (2004). Psycho-biological correlates of rapid ejaculation in patients attending an andrologic unit for sexual dysfunctions. Eur Urol..

[23] Strassberg DS, Mahoney JM, Schaugaard M, Hale VE (1990). The role of anxiety in premature ejaculation: a psychophysiological model. Arch Sex Behav.

[24] Barlow DH, Sakheim DK, Beck JG (1983). Anxiety increases sexual arousal. J Abnorm Psychol.

[25] Lewis RW (2001). Epidemiology of erectile dysfunction. Urol Clin North Am.

[26] Araujo AB, Durante R, Feldman HA, Goldstein I, McKinlay JB (1998). The relationship between depressive symptoms and male erectile dysfunction: cross-sectional results from the Massachusetts male aging study. Psychosom Med..

[27] Son H, Song SH, Lee JY, Paick JS (2011). Relationship between premature ejaculation and depression in Korean males. J Sex Med.

[28] Zhang X, Gao J, Liu J, Xia L, Yang J, Hao Z, Zhou J, Liang C (2013). Prevalence rate and risk factors of depression in outpatients with premature ejaculation. Biomed Res Int.

[29] Waldinger MD (2011). Toward evidence-based genetic research on lifelong premature ejaculation: a critical evaluation of methodology. Korean J Urol..

[30] Kalaitzidou I, Venetikou MS, Konstadinidis K, Artemiadis AK, Chrousos G, Darviri C (2014). Stress management and erectile dysfunction: a pilot comparative study. Andrologia.

[31] Sheehan DV, Lecrubier Y, Sheehan KH, Amorim P, Janavs J, Weiller E, Hergueta T, Baker R, Dunbar GC (1998). The Mini-International Neuropsychiatric Interview (M.I.N.I.): the development and validation of a structured diagnostic psychiatric interview for DSM-IV and ICD-10. J Clin Psychiatry.

[32] Rosen RC, Riley A, Wagner G, Osterloh IH, Kirkpatrick J, Mishra A (1997). The international index of erectile function (IIEF): a multidimensional scale for assessment of erectile dysfunction. Urology.

[33] Spielberger CD (1985). Assessment of state and trait anxiety: conceptual and methodological issues. South Psychol.

[34] Fountoulakis KN, Papadopoulou M, Kleanthous S, Papadopoulou A, Bizeli V, Nimatoudis I, Iacovides A, Kaprinis GS (2006). Reliability and psychometric properties of the Greek translation of the state-trait anxiety inventory form Y: preliminary data. Ann Gen Psychiatry.

[35] Beck AT, Ward CH, Mendelson M, Mock J, Erbaugh J (1961). An inventory for measuring depression. Arch Gen Psychiatry..

[36] Jemos J (1984). Beck depression inventory; validation in a Greek sample.

[37] Otto MW (1989). Anxiety and its disorders: the nature and treatment of anxiety and panic.

[38] McCabe MP (2005). The role of performance anxiety in the development and maintenance of sexual dysfunction in men and women. Int J Stress Manag.

[39] McCabe MP, Connaughton C (2013). Psychosocial factors associated with male sexual difficulties. J Sex Res.

[40] Barlow DH (1986). Causes of sexual dysfunction: the role of anxiety and cognitive interference. J Consult Clin Psychol.

[41] McCabe MP, Cobain MJ (1998). The impact of individual and relationship factors on sexual dysfunction among males and females. Sex Marital Ther.

[42] Janssen E, Everaerd W, Spiering M, Janssen J (2000). Automatic processes and the appraisal of sexual stimuli: toward an information processing model of sexual arousal. J Sex Res.

[43] Nobre PJ, Pinto-Gouveia J (2008). Cognitions, emotions, and sexual response: analysis of the relationship among automatic thoughts, emotional responses, and sexual arousal. Arch Sex Behav.

[44] Nobre PJ (2010). Psychological determinants of erectile dysfunction: testing a cognitive-emotional model. J Sex Med..

[45] Althof SE, Leiblum SR, Chevret-Measson M, Hartmann U, Levine SB, McCabe M, Plaut M, Rodrigues O, Wylie K (2005). Original research—psychology: psychological and interpersonal dimensions of sexual function and dysfunction. J Sex Med.

[46] Grupe DW, Nitschke JB (2013). Uncertainty and anticipation in anxiety: an integrated neurobiological and psychological perspective. Nat Rev Neurosci.

[47] Althof SE, Mcmahon CG, Waldinger MD, Serefoglu EC, Shindel AW, Adaikan PG, Becher E, Dean J, Giuliano F, Hellstrom WJG, Giraldi A, Glina S, Incrocci L, Jannini E, Mccabe M, Parish S, Rowland D, Segraves RT, Sharlip I, Torres LO (2014). An update of the International Society of Sexual Medicine’s Guidelines for the diagnosis and treatment of premature ejaculation (PE). J Sex Med.

[48] Waldinger MD (2007). Premature ejaculation: definition and drug treatment. Drugs.

[49] Carson C, Gunn K (2006). Premature ejaculation: definition and prevalence. Int J Impot Res..

[50] Dunn KM, Croft PR, Hackett GI (1999). Association of sexual problems with social, psychological, and physical problems in men and women: a cross sectional population survey. J Epidemiol Community Heal.

[51] Seidman SN (2002). Exploring the relationship between depression and erectile dysfunction in aging men. J Clin Psychiatry..

[52] Seidman SN, Roose SP (2000). The relationship between depression and erectile dysfunction. Curr Psychiatry Rep.

[53] Makhlouf A, Kparker A, Niederberger CS (2007). Depression and erectile dysfunction. Urol Clin North Am.

[54] Kennedy SH, Dugré H, Defoy I (2011). A multicenter, double-blind, placebo-controlled study of sildenafil citrate in Canadian men with erectile dysfunction and untreated symptoms of depression, in the absence of major depressive disorder. Int Clin Psychopharmacol.

[55] Nicolosi A, Moreira ED, Villa M, Glasser DB (2004). A population study of the association between sexual function, sexual satisfaction and depressive symptoms in men. J Affect Disord.

[56] Kantor J, Bilker WB, Glasser DB, Margolis DJ (2002). Prevalence of erectile dysfunction and active depression: an analytic cross-sectional study of general medical patients. Am J Epidemiol.

[57] Angst J (1998). Sexual problems in healthy and depressed persons. Int Clin Psychopharmacol.

[58] Shires A, Miller D (1998). A preliminary study comparing psychological factors associated with erectile dysfunction in heterosexual and homosexual men. Sex Marital Ther.

[59] Jannini EA, McMahon CG, Waldinger MD (2013). Premature ejaculation.

